# Risk factors for maintenance hemodialysis patients undergoing elective and emergency abdominal surgery

**DOI:** 10.1007/s00595-013-0828-6

**Published:** 2014-01-21

**Authors:** Hayato Abe, Ken-ichi Mafune

**Affiliations:** Division of Gastrointestinal Surgery, Mitsui Memorial Hospital, 1 Kandaizumichou, Chiyoda-ku, Tokyo, Japan

**Keywords:** Risk factor, Maintenance hemodialysis, Postoperative complication, Abdominal surgery

## Abstract

**Purpose:**

To identify the risk factors for morbidity and mortality after elective and emergency abdominal surgeries in maintenance hemodialysis patients.

**Methods:**

We retrospectively evaluated the medical records of 63 hemodialysis patients who underwent elective (group 1) and 24 who underwent emergency (group 2) abdominal surgeries, and classified them according to the presence/absence of postoperative complications. The clinical, laboratory and procedure-related data were obtained and compared between the groups.

**Results:**

Group 2 had significantly higher morbidity and mortality rates than group 1 (58.3 and 16.6 % vs. 33.3 and 16.6 %, respectively, *P* < 0.05). The patients in group 1 with and without complications had significantly different blood urea nitrogen (BUN) levels of 52.3 vs. 41.6 mg/dL (*P* = 0.03). There were significant differences in the patients in group 2 in terms of the age (72.7 vs. 55.0 years old; *P* < 0.002), the length of the operation (141 vs. 107 min; *P* < 0.02), the total protein levels (6.2 vs. 6.7 g/dL; *P* < 0.03), albumin levels (3.2 vs. 3.7 g/dL; *P* < 0.04) and need for intra- or postoperative blood transfusions (71.4 vs. 10.0 %; *P* < 0.005).

**Conclusions:**

The risk factors for a poor surgical outcome included high BUN levels in the elective surgery patients and hypoproteinemia, hypoalbuminemia, a longer operation and older age in patients undergoing emergency surgery. Perioperative blood transfusion was also associated with a high complication rate in the emergency surgery group.

## Introduction

Over 300,000 Japanese individuals annually undergo dialysis [1], and the number of patients receiving long-term hemodialysis is steadily increasing. Despite the well-described outcomes of end-stage renal disease (ESRD) among community-dwelling individuals, little is known regarding how long-term dialysis contributes to the risk of postoperative complications and mortality [2, 3]. Elective and emergency abdominal surgeries for gastrointestinal diseases are often required in patients receiving chronic hemodialysis. In addition to the usual considerations regarding perioperative management, a number of specific problems related to the surgical management of patients receiving hemodialysis exist. For example, surgery is associated with increased risks in these patients because of the presence of metabolic and coagulopathic disorders, as well as significant medical comorbidities that caused the renal failure [4]. Nevertheless, few reports exist on this increasingly important issue, particularly in the setting of emergency abdominal surgery in patients receiving maintenance hemodialysis at a single general hospital.

Therefore, this retrospective study aimed to identify the risk factors for morbidity and mortality after elective and emergency abdominal surgeries in patients on maintenance hemodialysis.

## Methods

We reviewed the medical records of 87 patients on maintenance hemodialysis who underwent abdominal surgery under general anesthesia at Mitsui Memorial Hospital (Tokyo, Japan) between January 2000 and December 2011. Patients who received either temporary dialysis for acute renal failure or peritoneal dialysis were excluded. The 87 patients were divided into elective (*n* = 63) or emergency (*n* = 24) surgery groups according to the type of surgical procedure performed. The elective and emergency surgery patients underwent hemodialysis 24–48 h after surgery depending on the blood urea nitrogen (BUN) and serum creatinine levels.

A total of 15 preoperative variables, including clinical, laboratory and procedure-related variables, were recorded for each patient. The clinical variables included age, gender, preoperative performance status (PS) immediately before surgery, diabetes and duration of maintenance hemodialysis. The laboratory variables included the preoperative BUN, serum creatinine, total protein, albumin and hemoglobin levels, and the hematocrit values. The procedure-related variables obtained from surgical records included the indications of preoperative cardiac complications and the need for pre-, intra- and postoperative blood transfusions in each group. Moreover, we considered whether diabetes was the cause of the renal failure in ESRD patients. All postoperative complications were identified through medical records. Surgical mortality was defined as an in-hospital death within 30 days after surgery, and the overall mortality included all hospital deaths.

Patients underwent either elective (group 1) or emergency (group 2) abdominal surgeries. The indications for elective surgery included esophageal cancer (*n* = 2), gastric cancer (*n* = 16), colorectal cancer (*n* = 32), liver cancer (*n* = 3), gallbladder cancer (*n* = 1), pancreatic cancer (*n* = 1) and cholelithiasis (*n* = 8). The indications for emergency surgery included upper gastrointestinal perforation (*n* = 3), lower gastrointestinal perforation (*n* = 6), appendicitis (*n* = 9), gastrointestinal bleeding (*n* = 2), inguinal hernia incarceration (*n* = 1), superior mesenteric artery thrombosis (*n* = 1), strangulated hernia (*n* = 1) and gallbladder hemorrhage (*n* = 1; Table 1).

**Table 1:** A variety of surgical procedures

Elective surgery	*n* = 63
Esophageal cancer	2
Gastric cancer	16
Colorectal cancer	32
Liver cancer	3
Gallbladder cancer	1
Pancreatic cancer	1
Cholelithiasis	8
Emergency surgery	*n* = 24
Upper GI perforation	3
Lower GI perforation	6
Appendicitis	9
GI bleeding	2
Inguinal hernia incarceration	1
SMA thrombosis	1
Strangulated hernia	1
Gallbladder bleeding	1

*GI* gastrointestinal, *SMA* superior mesenteric artery

The values are presented as the mean ± SD. The differences in categorical variables between groups were analyzed using the Chi squared test, and those between continuous variables were analyzed using the Mann–Whitney *U* test. A probability (*P*) value <0.05 was considered to be statistically significant. All analyses were performed using the JMP^®^ v9 statistical software program (SAS Institute Inc., Cary, NC, USA).

## Results

The overall morbidity and mortality rates were 41.4 % (36/87) and 5.7 % (5/87), respectively, in groups 1 and 2. In group 1, wound infection and dehiscence (29 %), followed by abdominal abscess (14 %), cardiac disease (14 %), pulmonary disease (14 %) and anastomotic leakage (9.5 %), were the most frequently observed complications. In group 2, wound infection and dehiscence (64 %), followed by cerebrovascular disease (14 %), sepsis (14 %) and intestinal bleeding (7.1 %), were the most frequently observed complications (Table 2). The morbidity and mortality rates were 33.3 and 1.5 % in group 1 and 58.3 and 16.6 % in group 2, respectively (*P* < 0.05 and <0.02, respectively). Of the five perioperative deaths, one patient who underwent elective surgery died of cardiac failure caused by pericarditis, and four patients who underwent emergency surgery died of multiple organ failure, sepsis, peritonitis, cerebral infarction, acute subdural hematoma and/or intestinal bleeding (Table 3).

**Table 2:** Postoperative complications following abdominal surgery in patients undergoing hemodialysis

	*n* (%)
Number of patients with complications	35 (40)^a^
Number of complications	37
Type and number of patients with each complication	
Elective group (*n* = 63)	21
Wound infection/dehiscence	5/1 (29)^b^
Abdominal abscess	3 (14)^b^
Cardiac disease	3 (14)^b^
Pulmonary disease	3 (14)^b^
Cerebral vascular disease	1 (4.8)^b^
Anastomotic leakage	2 (9.5)^b^
Intestinal obstruction	1 (4.8)^b^
Pancreatic fistula	1 (4.8)^b^
Others	2 (9.5)^b^
Emergency group (*n* = 24)	14
Wound infection/dehiscence	8/1 (64)^b^
Cerebral vascular disease	2 (14)^b^
Sepsis	2 (14)^b^
Intestinal bleeding	1 (7.1)^b^
Others	1 (7.1)^b^

^a^Of a total of 87 patients

^b^The ratio against the number of patients with complications in each group

**Table 3:** Patients who died of disease related to an operative complication

Case	Age (years)/sex	Elective or emergency	Diagnosis	Operation method	Operation time (min)	Amount of bleeding (mL)	Post operative complication	Total protein (g/dL)	Cardiovascular disorder	Intra- and postoperative blood transfusion	Death of operations (days)
1	62/F	Elective	Gastric cancer	Total gastrectomy	253	447	Pericarditis	5.8	–	+	14
2	58/M	Emergency	SMA thrombosis	Bowel resection	96	81	Small intestinal bleeding	6.3	+	+	4
3	75/M	Emergency	Small intestinal perforation	Bowel resection	97	10	Cerebral infarction	7.6	+	–	1
4	86/F	Emergency	Diverticula perforation of colon	Partial colectomy + colostomy	207	220	DIC/Sepsis/MOF	6.9	–	+	6
5	75/M	Emergency	Gallbladder bleeding	Cholecystectomy	117	870	Acute subdural hematoma	6.1	+	+	11

With regard to the preoperative, clinical and laboratory variables, there were significant differences between the patients in each group in the preoperative PS (1.09 vs. 2.58, *P* < 0.0001) and albumin levels (3.7 vs. 3.4 g/dL, *P* < 0.05), but there were no significant differences among the other variables (Table 4).

**Table 4:** Preoperative clinical and laboratory characteristics in elective and emergency surgery groups

Variable	Elective surgery (*n* = 63)	Emergency surgery (*n* = 24)	*P* value
Age (years)	66.2 ± 8.2	65.3 ± 13.6	0.64^a^
Sex (male/female)	44/19	15/9	0.60^b^
Performance status	1.09 ± 1.01	2.58 ± 0.58	0.0001^a^
Time on hemodialysis (months)	95.2 ± 92.4	126 ± 111	0.21^a^
Blood urea nitrogen (mg/dL)	45.2 ± 17.0	49.5 ± 16.3	0.25^a^
Creatinine (mg/dL)	8.2 ± 2.1	8.5 ± 2.1	0.70^a^
Total protein (g/dL)	6.5 ± 0.7	6.4 ± 0.5	0.35^a^
Albumin (g/dL)	3.7 ± 0.4	3.4 ± 0.7	0.042^a^
Hemoglobin (g/dL)	9.7 ± 1.4	10.1 ± 2.2	0.56^a^
Hematocrit (%)	30.0 ± 4.5	30.7 ± 5.6	0.67^a^
Cardiovascular disorders (%)	36.5	20.8	0.20^b^
Diabetes mellitus required treatment (%)	22.2	4.1	
Hemodialysis induced by diabetes (%)	49.2	16.6	

Values are shown as mean ± SD; otherwise, number of patients or percentage in patients are shown

*P* < 0.05 was considered significant

*P* values were calculated using ^a^the Mann–Whitney *U* test or ^b^the Fisher’s exact test

With regard to the clinical, laboratory and procedure-related variables, significant differences were found only in the BUN levels (52.3 vs. 41.6 mg/dL, respectively, *P* = 0.03) between patients with and without postoperative complications in group 1. However, in group 1, no significant differences were found between the patients with and without complications in any of the other variables examined, including the age, gender ratio, mean duration of preoperative hemodialysis, preoperative PS, cardiovascular disorders, diabetes, diabetes-induced hemodialysis, serum creatinine, albumin and hemoglobin levels, the hematocrit value or the need for pre-, intra- or postoperative blood transfusions (Table 5).

**Table 5:** Clinical and laboratory characteristics of 63 hemodialysis patients undergoing elective abdominal surgery

Variable	With complications (*n* = 21)	Without complications (*n* = 42)	*P* value
Age (years)	65.1 ± 6.6	66.7 ± 8.8	0.45^a^
Sex (male/female)	16/5	28/14	0.56^b^
Performance status	0.42 ± 0.59	0.69 ± 0.89	0.23^a^
Time on hemodialysis (months)	80.2 ± 85.4	87.9 ± 97.2	0.94^a^
Operation time (min)	231 ± 188	182 ± 68	0.19^a^
Amount of bleeding (mL)	234 ± 215	245 ± 260	0.87^a^
Blood urea nitrogen (mg/dL)	52.3 ± 21.3	41.6 ± 13.3	0.030^a^
Creatinine (mg/dL)	8.2 ± 2.3	8.2 ± 2.1	0.96^a^
Total protein (g/dL)	6.3 ± 0.9	6.6 ± 0.6	0.25^a^
Albumin (g/dL)	3.6 ± 0.5	3.7 ± 0.4	0.53^a^
Hemoglobin (g/dL)	9.7 ± 1.6	9.7 ± 1.4	0.91^a^
Hematocrit (%)	30.1 ± 5.2	30.0 ± 4.1	0.93^a^
Cardiovascular disorders (%)	52.4	28.6	0.095^b^
Diabetes mellitus required treatment (%)	36.3	14.6	0.052^b^
Hemodialysis induced by diabetes (%)	52.4	50.0	0.99^b^
Blood transfusion (%)			
Perioperative	47.6	31.0	0.26^b^
Preoperative	28.6	21.4	0.54^b^
Intra- and postoperative	33.3	14.3	0.10^b^

Values are shown as mean ± SD; otherwise, number of patients or percentage in patients are shown

*P* < 0.05 was considered significant

*P* values were calculated using ^a^the Mann–Whitney *U* test or ^b^the Fisher’s exact test

In group 2, the patient age (72.7 vs. 55.0 years, *P* < 0.002), duration of surgery (141 vs. 107 min, *P* < 0.02), total protein level (6.2 vs. 6.7 g/dL, *P* < 0.03), albumin level (3.2 vs. 3.7 g/dL, *P* < 0.04) and the need for intra- and postoperative blood transfusion (71.4 vs. 10.0 %, *P* < 0.005) were significantly different between the patients with and without complications (Table 6).

**Table 6:** Clinical and laboratory characteristics of 24 hemodialysis patients undergoing emergency abdominal surgery

Variable	With complications (*n* = 14)	Without complications (*n* = 10)	*P* value
Age (years)	72.7 ± 8.7	55.0 ± 12.8	0.0017^a^
Sex (male/female)	8/6	7/3	0.67^b^
Performance status	1.0 ± 1.0	0.4 ± 0.6	0.10^a^
Time on hemodialysis (months)	109 ± 112	151 ± 111	0.42^a^
Operation time(min)	141 ± 36.9	107 ± 44	0.022^a^
Amount of bleeding (mL)	187 ± 242	58 ± 58	0.12^a^
Blood urea nitrogen (mg/dL)	54.5 ± 19.7	42.5 ± 10.2	0.083^a^
Creatinine (mg/dL)	7.8 ± 1.8	9.4 ± 2.3	0.089^a^
Total protein (g/dL)	6.2 ± 0.6	6.7 ± 0.4	0.027^a^
Albumin (g/dL)	3.2 ± 0.7	3.7 ± 0.5	0.037^a^
Hemoglobin (g/dL)	9.5 ± 1.8	10.9 ± 2.4	0.18^a^
Hematocrit (%)	29.8 ± 5.8	32.0 ± 5.3	0.41^a^
Cardiovascular disorders (%)	28.5	10.0	0.35^b^
Diabetes mellitus required treatment (%)	0.0	10.0	0.41^b^
Hemodialysis induced by diabetes (%)	21.4	10.0	0.61^b^
Blood transfusion (%)			
Perioperative	71.4	10.0	0.0045^b^
Preoperative	21.4	0.0	0.23^b^
Intra- and postoperative	71.4	10.0	0.0045^b^

Values are shown as mean ± SD; otherwise, number of patients or percentage in patients are shown

*P* < 0.05 was considered significant

*P* values were calculated using ^a^the Mann–Whitney *U* test or ^b^the Fisher’s exact test

## Discussion

In recent years, the number of ESRD patients requiring long-term dialysis has been increasing. For this reason, the number of surgeries performed in patient requiring dialysis has increased. There are many problems influencing the outcome of surgical care in patients receiving hemodialysis, including the prevalence of cardiovascular disease, atherosclerotic heart disease, diabetes, hypertension, hypotension, electrolyte imbalances, acidosis, hematopoietic disorders, coagulation disorders, infections and impaired wound healing. Therefore, these problems must be preoperatively addressed, and special care needs to be taken postoperatively, including re-initiating hemodialysis [4]. Patients on maintenance hemodialysis undergoing abdominal surgery frequently experience postoperative complications, which often lead to death [5].

In the present study, the overall complication and mortality rates in patients with abdominal surgery were 41.8 and 5.7 %, respectively. The complication and mortality rates were 33.3 and 1.5 % in group 1, and 58.3 and 16.6 % in group 2, respectively, with a significantly higher mortality rate in group 2. These data are comparable to the findings of other reports [6–8]. In the patients undergoing emergency surgery, bacteremia was presumed to be the cause of postoperative sepsis or disseminated intravascular coagulation that resulted in multiple organ failure. Furthermore, the production of cytokines or reactive oxygen species caused by preoperative infections, which did not seem to be eliminated by preoperative hemodialysis, could have negatively influenced the treatment outcomes [6].

Regarding the preoperative clinical and laboratory characteristics, the PS in group 2 was worse than that in group 1. The presence of hypoalbuminemia was attributed to the increased utilization of serum albumin due to acute inflammatory reactions. In each group, postoperative wound-related complications, such as wound infection and dehiscence, were the most frequent complications. Previous studies have documented similarly high incidences of postoperative wound complications in dialysis patients [6–8]. First, wound-related complications are influenced by a delay in wound healing in patients on maintenance hemodialysis [7]. Second, many infectious complications, such as abdominal abscesses, pulmonary complications and sepsis, were observed in these patients. For example, Drolet et al. [9] reported a 52 % incidence of complications in dialysis patients, 25 % of whom developed infectious postoperative complications. Infections and wound healing complications were most likely associated with uremia. A high BUN concentration is often considered to be a characteristic of latent uremia due to infrequent hemodialysis [4], and the laboratory data analysis revealed that postoperative complications were associated with a high BUN concentration in patients following elective abdominal surgery. Therefore, addressing these issues through planned preoperative hemodialysis can contribute to decreasing the morbidity and mortality rates [7]. In this study, the elective surgery patients received hemodialysis within 24 h before surgery; however, the patients who underwent emergency surgery did not receive preoperative hemodialysis.

Although the precise reason for the high incidence of pulmonary complications in patients receiving hemodialysis remains unknown, some studies have speculated that dialysis may induce hypoxemia because of carbon dioxide diffusion through the dialysate, subsequently leading to hypocapnia and reflex hypoventilation [9–11]. Although the percentage of patients who died from fatal cardiac or cerebrovascular complications was high, we suspect that these complications were likely caused by systemic vascular atherosclerosis in those receiving maintenance hemodialysis.

In addition, the laboratory data analysis revealed that postoperative complications were associated with low total protein and albumin levels, a longer operation and an older age in patients who underwent emergency abdominal surgery. In emergency surgery, the fact that the patients received intra- and postoperative blood transfusions suggested that they had anemia, low total protein and albumin levels, or decreased concentrations of coagulation factors. Although chronic anemia accompanying hemodialysis is well tolerated by patients receiving maintenance hemodialysis, preoperative malnutrition and increased utilization of the serum protein and albumin which occurred due to acute inflammatory reactions following surgery, were significant risk factors for poor outcomes of emergency abdominal surgery. Moreover, in patients undergoing maintenance hemodialysis, it is necessary to consider the risk of the hemodynamic load placed on the circulatory system by the increase in water volume caused by blood transfusion.

The principal aims of preoperative dialysis are to ensure maximum metabolic control and to avoid fluid overload, hyperkalemia and excessive bleeding. Consequently, many problems observed in our patients had been preoperatively addressed to a reasonable degree, and all who underwent elective surgery received hemodialysis before surgery to help decrease the risks. The most common indication for an early initiation of dialysis following surgery was increased serum potassium levels, which were often elevated by infection, catabolic disease, blood transfusions and intraoperative tissue trauma; therefore, repeated dialysis sessions were deemed sufficiently manageable [4]. In our study, all elective surgery patients received hemodialysis on the second postoperative day to maintain normal electrolyte levels and avoid volume overload.

Diabetes mellitus is the most common cause of renal failure in ESRD patients, with a reported rate of 44.3 % of those receiving chronic hemodialysis [1]. Moreover, diabetes is associated with pulmonary and other postoperative complications, such as myocardial infarction and stroke [8]. Although the need for hemodialysis was higher in patients with complicated diabetes, this factor did not show statistical significance in this study.

In conclusion, the morbidity and mortality rates were high following abdominal surgery in patients receiving maintenance hemodialysis, particularly in those who underwent emergency surgery. The risk factors for poor surgical outcomes were high BUN levels in the elective surgery patients, and hypoproteinemia, hypoalbuminemia, a longer operation and an older age in the patients who underwent emergency surgery. Perioperative blood transfusion, particularly following intra- and postoperative blood transfusion, was associated with high complication rates in the emergency surgery patients. However, further studies with larger patient cohorts are required to confirm our findings and to establish guidelines for perioperative care after abdominal surgery in patients on maintenance hemodialysis.
